# An Efficient Wi-Fi Sensing Method for Robotic Arm Motion Recognition

**DOI:** 10.3390/s26103210

**Published:** 2026-05-19

**Authors:** Junyan Zhuo, Qingrui Wang, Yuzhou Sheng, Xi Wang, Yuxuan Zhang, Xiaojing Wan

**Affiliations:** School of Mechanical Engineering, Xinjiang University, Urumqi 830049, China; zhuojunyan3@gmail.com (J.Z.); 14769974985@163.com (Q.W.); claouaywhyeyskusyahwywf@gmail.com (Y.S.); salanitrivanesarock122@gmail.com (X.W.); 13565870617@163.com (Y.Z.)

**Keywords:** channel state information, robotic arm motion recognition, lightweight transformer, adaptive temporal downsampling, temporal gating module

## Abstract

In recent years, channel state information (CSI)-based sensing technology has gradually attracted widespread attention as a contactless and low-cost approach for robotic arm motion understanding. Despite continuous progress in CSI-based human sensing, existing methods of robotic motion sensing still face two key challenges when directly applied to robotic motion sensing: (1) CSI perturbations induced by robotic arm motion are weak and locally distributed, making fine-grained feature extraction difficult. (2) Discriminative information in long robotic arm motion sequences is sparsely concentrated in a few key intervals, and its adaptive temporal selection and enhancement remain challenging. To address the above challenges, this paper proposes an efficient multi-stage robotic arm motion recognition method (named MSPoolNet). The proposed method consists of three key modules: an adaptive temporal downsampling module, a temporal gating module, and a Transformer-based feature encoding module. Specifically, the adaptive temporal downsampling module processes the raw CSI signal at the input stage to achieve local pattern extraction. The temporal gating module adaptively reweights temporal features, dynamically highlighting key temporal segments while suppressing irrelevant information. The proposed Transformer-based feature encoding module replaces conventional self-attention with pooling operations, enabling global information interaction and fine-grained feature representation in a computationally efficient manner. Extensive experimental results demonstrate that the proposed method achieves state-of-the-art performance on two representative public datasets, maintaining a compact model size with an accuracy exceeding 99%.

## 1. Introduction

With the rapid development of Industry 4.0 and intelligent manufacturing, accurate perception of robotic arm motion has become increasingly important in application scenarios such as automated production, human–robot collaboration, and intelligent control. Existing sensing methods mainly rely on vision-based methods or additional sensors. However, vision-based methods are susceptible to factors such as illumination changes and target occlusion, which limits their applicability. Sensor-based solutions usually require additional hardware deployment and maintenance costs and may interfere with the normal operation of the equipment to a certain extent. Compared with the above two sensing approaches, CSI-based sensing technology can leverage existing wireless communication infrastructure to obtain robotic arm motion information in a low-cost and non-intrusive manner, showing promising application potential in the field of robotic arm monitoring. Nevertheless, in robotic arm motion recognition, extracting discriminative features of different motions from CSI signals still faces the following challenges.

First, signal perturbations of CSI caused by robotic arm motion are weak and locally distributed, making effective features difficult to capture adequately. In practical deployment environments, the limited deployment space and the physical dimensions of the robotic arm usually lead to small and localized CSI fluctuations during motion. Such subtle signal perturbations are easily masked by environmental noise and changes in measurement conditions. In addition, robotic arm movements are usually highly repetitive and continuous, which makes the differences among different motions even more difficult to distinguish under noisy conditions. Therefore, it is necessary for a robotic arm motion recognition framework to remain sensitive to weak local patterns and extract discriminative features robustly under noisy conditions to accurately distinguish fine-grained motions.

Second, in CSI signals generated by continuous robotic arm motion, discriminative information is often concentrated in only a few key periods, making adaptive extraction of these critical temporal features another major challenge. Specifically, within a fixed-length CSI acquisition window, the channel variations that truly reflect motion differences usually appear only in a few key temporal segments, while the remaining periods mainly correspond to stable background or weakly correlated responses. If all temporal positions are assigned equal weights, useful discriminative information may be diluted by redundant signals, thereby reducing recognition accuracy. Moreover, the key motion segments of different motions may vary considerably in both length and occurrence time, which further increases the difficulty of temporal modeling. Therefore, it is important for a robotic arm motion recognition model to automatically identify key periods and dynamically enhance discriminative information.

In addition, Wi-Fi-based robotic arm motion recognition can also be influenced by deployment conditions, multipath propagation, and motion speed variations, which further increase the practical difficulty of CSI-based representation learning.

To address the above challenges, this paper proposes MSPoolNet, a multi-stage CSI-based model for robotic arm motion recognition. The proposed framework is developed to better accommodate weak local CSI perturbations and sparsely distributed informative temporal cues, while preserving a favorable balance between recognition performance and computational efficiency. The main contributions of this paper can be summarized as follows:

- We propose an efficient CSI-based robotic arm motion recognition model, MSPoolNet, which consists of three key modules: an adaptive temporal downsampling module, a temporal gating module, and a Transformer-based feature encoding module. Specifically, the adaptive temporal downsampling module is used to extract local time–frequency patterns at the input stage. The temporal gating module can dynamically highlight key temporal segments and enhance the representation capability of discriminative features. The Transformer-based feature encoding module achieves global temporal modeling of fine-grained features while maintaining low computational overhead.
- We propose a fine-grained feature extraction module based on CSI, which integrates a lightweight Transformer encoding structure and replaces conventional self-attention with pooling operations, thereby enhancing the modeling of subtle temporal dependencies and fine-grained discriminative patterns in CSI signals while achieving an effective balance between computational efficiency and representation capability.
- We conduct extensive experiments on two public CSI datasets for robotic arms. The results demonstrate that the proposed method achieves state-of-the-art performance. Furthermore, comparative experiments further verify its applicability to related robotic arm motion recognition tasks.

## 2. Related Work

### 2.1. Wi-Fi Sensing for Human Activity Recognition

In recent years, human activity recognition based on CSI has attracted widespread attention and has gradually expanded to a variety of tasks, including gesture recognition, fall detection, and pose sensing [1,2]. Existing studies have shown that Wi-Fi signals are affected by multipath phenomena such as reflection, scattering, and diffraction during propagation, and the received signals exhibit corresponding amplitude and phase variations when the human body moves within the environment. Compared with received signal strength indication (RSSI), which only provides coarse-grained signal strength information, CSI can characterize more fine-grained time-varying channel responses at the level of orthogonal frequency division multiplexing (OFDM) subcarriers and has therefore gradually become an important information carrier for contactless behavior sensing [3,4]. Existing studies have further indicated that this field has developed along a relatively clear trajectory, evolving from early methods based on wireless propagation mechanism analysis and hand-crafted feature extraction to end-to-end modeling paradigms centered on deep learning [2,3].

From the perspective of methodological development, research on Wi-Fi CSI-based human activity recognition has evolved from early signal propagation modeling to deep learning-based representation learning frameworks. Early studies mainly relied on explicit modeling of wireless propagation characteristics to support activity recognition and tracking. For example, Wang et al. investigated human activity recognition by analyzing multipath propagation effects and CSI variations, while Adib et al. demonstrated the feasibility of tracking human motion through radio reflections [5,6,7]. Concurrently, several pioneering systems demonstrated the feasibility of Wi-Fi-based gesture and activity recognition, including WiSee [8] for whole-home gesture recognition, WiGest [9] for ubiquitous gesture sensing, and E-eyes [10] for device-free activity identification. As sensing tasks became more complex, research gradually shifted toward data-driven approaches. In this direction, Wang et al. introduced CNN-based modeling to learn local patterns from CSI representations [11], whereas Chen et al. employed an attention-based BLSTM framework to capture temporal dependencies in passive human activity recognition [12]. Subsequently, Kang et al. further improved the efficiency of sequence modeling by developing a lightweight attention GRU architecture for Wi-Fi CSI-based human activity recognition [13] and Ma et al. demonstrated the effectiveness of Wi-Fi sensing in sign language recognition [14]. In addition, Zhou et al. explored signal-to-image transformation to enable fine-grained gesture recognition from commercial Wi-Fi devices [15] and Zheng et al. proposed Widar3.0 for cross-domain gesture recognition with Wi-Fi [16]. More recently, the introduction of attention mechanisms and Transformer-based architectures has further strengthened the ability of Wi-Fi sensing models to capture long-range dependencies and complex dynamic patterns. Related advances can be observed in the studies of Hu et al., Vaswani et al., Dosovitskiy et al., and Yang et al. [17,18,19,20].

However, most of the above studies were designed for human-scale motion sensing tasks, whereas robotic arm motion usually induces weaker and more localized CSI variations. Therefore, although human activity recognition research has provided an important methodological foundation for Wi-Fi sensing, existing methods cannot be directly transferred to robotic arm behavior recognition, and more task-specific designs are still required.

### 2.2. Wi-Fi Sensing for Robotic Arm Motion Recognition

Compared with human activity recognition, research on robotic arm motion recognition based on CSI remains relatively limited and is still at an early stage. Representative studies mainly include RoboFiSense and RoboMNIST. The former focuses on robotic arm motion recognition and systematically investigates recognition performance under different receiver placements, motion speeds, and device configurations, thereby providing an early public evaluation framework for Wi-Fi-based robotic arm sensing [21]. Building on this line of research, RoboMNIST further extends the conventional handwritten digit recognition paradigm to robotic arm scenarios. By formulating digit-writing tasks, it advances the problem setting from coarse-grained activity recognition to finer-grained motion discrimination [22]. In addition, the platform incorporates not only CSI but also multimodal information such as video and audio, making its task formulation and data organization more comprehensive than those of earlier studies. As a result, compared with prior work that mainly emphasized the recognizability of robotic arm activities, this benchmark places greater emphasis on distinguishing subtle motion differences and thus imposes more demanding evaluation criteria on CSI-based robotic arm motion recognition.

The methodological development of CSI-based robotic arm motion recognition has gradually progressed from early exploratory modeling to more sophisticated deep neural network architectures. Early studies employed convolutional neural networks to model CSI sequences induced by robotic arm movements, achieving recognition of multiple activity categories for the Franka Emika robotic arm and preliminarily validating the feasibility of CSI for robotic motion perception [23]. Subsequently, RoboFiSense introduced BiVTC, which incorporated a dual-branch Vision Transformer into robotic arm Wi-Fi sensing, performing dual-path encoding and feature concatenation on CSI features to achieve robotic arm motion recognition under different receiver placements and motion speed settings [21]. Following this line of research, subsequent studies explored the combination of wavelet transforms and Vision Transformers to enhance the time–frequency representation of CSI and further improve robotic motion recognition performance [24]. In addition, RoboDSTAN adopted a dual-stream temporal convolution architecture combined with attention mechanisms to model robotic arm motion [25]. More recently, research has begun to extend beyond amplitude-based modeling to consider CSI phase information. For example, Zandi et al. [26] proposed a GF-BiLSTM framework based on amplitude–phase dual-stream gated fusion, demonstrating that the joint utilization of amplitude and phase can further improve recognition accuracy and robustness under cross-speed evaluation settings. Overall, existing research has evolved from early CNN-based modeling to Transformer-based architectures, dual-stream temporal modeling, and amplitude–phase joint modeling, thereby broadening the methodological scope of CSI-based robotic arm motion recognition.

Nevertheless, existing studies still leave room for improvement. Most current work remains focused on robotic arm activity classification, while frameworks specifically designed for finer-grained motion discrimination are still limited. In addition, representative methods mainly build on general purpose architectures, rather than establishing a compact and task-oriented modeling pipeline tailored to robotic arm CSI signals. Therefore, CSI modeling for robotic arm motion recognition still calls for a dedicated framework that can jointly support compact front-end representation, progressive information reorganization, and efficient back-end encoding, which also constitutes the direct motivation for the method proposed in this paper.

## 3. Proposed Method

### 3.1. Problem Formulation

In the robotic arm Wi-Fi sensing scenario considered in this study, a wireless transmitter and multiple passive receivers are deployed around the robotic workspace to capture channel variations induced by arm motion, without requiring attached sensors or vision equipment. As the robotic arm performs different motion patterns, the transmitted Wi-Fi signals undergo multipath propagation, and the resulting channel state information is recorded by the receivers over successive packets and subcarriers. These CSI measurements provide a non-intrusive representation of robotic motion and serve as the sensing basis for subsequent recognition.

In an OFDM-based Wi-Fi system, the received signal can be written as

(1) y=Hx+η,

where x, y, and η denote the transmitted signal, received signal, and additive noise, respectively [21,22]. The CSI matrix $H\in C^{T\times S}$ characterizes the channel response over *T* packets and *S* subcarriers, and each element is defined as

(2) $$h_{s}\left[t\right]=a_{s}\left[t\right]\;e^{j\phi_{s}\left[t\right]},$$

where $a_{s}\left[t\right]$ and $\phi_{s}\left[t\right]$ denote the amplitude and phase of subcarrier *s* at time index *t*. This study uses CSI amplitude as the input feature. This choice follows the common setting adopted in many CSI-based recognition studies, where amplitude provides a comparatively stable and reproducible representation. In contrast, the raw CSI phase is highly sensitive to hardware-induced offsets and usually requires additional calibration procedures, such as phase unwrapping, sanitization, and offset removal. These procedures introduce extra preprocessing complexity and computational cost, which is less consistent with the lightweight design goal of MSPoolNet. Therefore, this work focuses on amplitude-based robotic arm motion recognition, while phase-amplitude fusion is left for future investigation.

Given a CSI amplitude matrix A, the objective of the classification task is to infer the activity label y∈{1,2,…,C} corresponding to the observed channel variations. Formally, let ${\left\{\left(A_{i},y_{i}\right)\right\}}_{i=1}^{N}$ denote the labeled training set, where $A_{i}\in R^{T\times S}$ represents the CSI amplitude matrix and $y_{i}$ denotes the corresponding class label. The goal of this study is to learn a parametric mapping $f_{\theta }:R^{T\times S}\to \left\{1,\ldots ,C\right\}$ by minimizing the cross-entropy loss over the training set.

### 3.2. Proposed Framework

MSPoolNet is developed as a multi-stage framework for robotic arm motion recognition from Wi-Fi CSI. Given a raw CSI amplitude matrix $A\in R^{1\times T\times S}$, the framework consists of six sequential stages: signal preprocessing, an adaptive temporal downsampling module, a temporal gating module, a patch embedding module, a Transformer-based feature encoding module, and a classification head. Through this hierarchical design, the input is progressively transformed from normalized CSI representations into compact and discriminative features for final motion recognition.

In robotic arm CSI sensing, discriminative information is often subtle and tends to appear as localized variations in time–frequency responses. Moreover, useful cues are unevenly distributed along the temporal dimension, since a fixed-length acquisition window usually contains a considerable proportion of stable background segments and weakly correlated disturbances. Practical deployment also requires an effective balance between parameter efficiency and inference performance. To accommodate these characteristics, MSPoolNet first performs signal preprocessing to obtain a normalized CSI representation. It then employs an adaptive temporal downsampling module to enhance local time–frequency patterns while reducing frequency-domain redundancy, uses a temporal gating module to emphasize informative CSI responses, and adopts a Transformer-based feature encoding module to model compact token representations efficiently.

**Figure 1:**
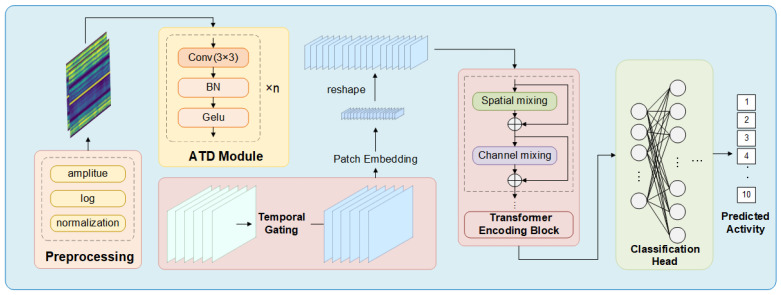
Overall architecture of the proposed MSPoolNet for CSI-based robotic arm motion recognition. The raw CSI amplitude matrix is first processed by the signal preprocessing stage, and the resulting representation is then fed into the adaptive temporal downsampling module, temporal gating module, patch embedding, and lightweight Transformer encoding blocks, followed by global average pooling and a linear classifier.

Accordingly, MSPoolNet is organized as a progressive pipeline comprising input preprocessing, local pattern extraction, key temporal interval selection, and high-level representation integration. This design is specifically tailored to robotic arm CSI signals, including subtle local disturbances, sparsely distributed informative temporal cues, and the practical need for efficient back-end modeling. The overall architecture is shown in Figure 1, and the key components are described in the following subsections.

#### 3.2.1. Signal Preprocessing

The raw CSI data is stored in the form of a complex matrix $H\in C^{T\times S}$. The amplitude matrix $M\in R^{T\times S}$ is extracted from H as

(3) $$M_{t,s}=\left|h_{s}\left[t\right]\right|.$$

Log compression is applied to reduce the dynamic range:

(4) $${\tilde{A}}_{t,s}=\log\left(M_{t,s}+\epsilon \right),\;\epsilon =10^{-6}.$$

The compressed amplitude is further normalized along each subcarrier:

(5) $${\hat{A}}_{t,s}=\frac{{\tilde{A}}_{t,s}-\mu_{s}}{\sigma_{s}+\epsilon }.$$

Here, $\mu_{s}$ and $\sigma_{s}$ denote the mean and standard deviation of subcarrier *s* over the temporal dimension within the same CSI sample, respectively.

#### 3.2.2. Adaptive Temporal Downsampling Module

To better preserve weak and locally distributed motion-related perturbations in robotic arm CSI, MSPoolNet employs an adaptive temporal downsampling module as the front-end representation stage. By enhancing local time–frequency responses and reducing redundant subcarrier information before tokenization, this module produces a compact feature representation for subsequent temporal gating and encoding. This design is supported by recent findings showing that early convolutional layers can improve both the optimization process and the feature quality of Transformer architectures [27]. The module corresponds to the overall mapping process defined in Equation (6), and its primary roles are to extract local time–frequency patterns and perform early-stage resolution reduction.

The adaptive temporal downsampling module is composed of two convolutional layers, each followed by batch normalization and a nonlinear activation function [28]. Considering that the discriminative information of robotic arm motions in CSI is primarily distributed along the temporal dimension, while adjacent subcarriers in the frequency dimension often exhibit strong correlation and redundancy, an asymmetric downsampling strategy is adopted, with relatively moderate compression in the temporal dimension and more aggressive compression in the frequency dimension. Specifically, the first layer performs joint downsampling in both time and frequency, whereas the second layer further reduces the frequency resolution. This design aims to preserve the dynamic boundaries and temporal evolution of the motion as much as possible, thereby avoiding excessive loss of temporal detail at an early stage, while simultaneously exploiting the redundancy among adjacent subcarriers to reduce the input scale for subsequent encoding.

For robotic arm motion recognition, inter-class differences are usually not reflected in large-scale global shape variations but rather in subtle local response differences across neighboring time steps and adjacent subcarriers, such as short-term energy fluctuations, local amplitude perturbations, and variations in inter-subcarrier correlation patterns. Therefore, the adaptive temporal downsampling module in the proposed model is not merely used for general feature extraction but is designed to enhance local spatiotemporal responses in raw CSI at an early stage so that discriminative cues related to fine-grained motion differences can be more effectively preserved.

Let the preprocessed CSI tensor be $\hat{A}\in R^{1\times T_{in}\times F_{in}}$. To align the feature size for subsequent patch embedding, zero-padding is applied along the frequency dimension to obtain ${\hat{A}}_{pad}\in R^{1\times T_{in}\times F_{pad}}$. The stem output is defined as

(6) $$F_{stem}=\mathrm{Stem}\left({\hat{A}}_{pad}\right)\in R^{C_{stem}\times T_{stem}\times F_{stem}}.$$

Here, $C_{stem}$ denotes the number of output channels, while $T_{stem}$ and $F_{stem}$ represent the downsampled temporal and frequency resolutions, respectively. The adaptive temporal downsampling module maps the original CSI input into a more compact, multi-channel feature representation, thereby establishing the feature basis for subsequent temporal gating and token-based modeling. The two-layer Conv-BN-GELU cascaded structure of the adaptive temporal downsampling module and its asymmetric time–frequency downsampling scheme are illustrated in Figure 1.

#### 3.2.3. Temporal Gating Module

The CSI features produced by the adaptive temporal downsampling module remain continuous along the temporal dimension, whereas the truly informative discriminative cues associated with robotic arm motions are typically concentrated within only a few key temporal intervals. Within a fixed-length CSI acquisition window, stable background segments before and after motion onset, transition periods, and weak disturbance regions often occupy a substantial portion of the sequence. In contrast, the channel variations that genuinely reflect motion differences are usually short-lived and appear at uncertain temporal locations. If these informative intervals are not explicitly highlighted, the subsequent encoder must process a large amount of weakly correlated or irrelevant CSI responses, thereby increasing the difficulty of accurately identifying critical motion-related segments. To better focus the representation on motion-relevant temporal cues, the proposed framework employs a temporal gating module to perform sample-dependent feature reweighting along the temporal dimension.

The temporal gating module compresses the convolutional features along the channel and frequency dimensions into a one-dimensional temporal descriptor, which characterizes the overall response intensity of the CSI sequence at different time positions. Based on this descriptor, a multi-layer perceptron (MLP) predicts time-step-wise gating weights in the range of 0 to 1, and these weights are then applied to reweight the convolutional features. In this way, the module emphasizes informative channel responses associated with robotic arm motions while preserving the original temporal order, thereby suppressing the influence of stable background segments and weakly correlated disturbances during subsequent tokenization and encoding.

This module consists of three steps. First, the output F from the adaptive temporal downsampling module is averaged across the channel and frequency dimensions, compressing each time step into a scalar to obtain a temporal vector that reflects the overall activity intensity at each time step:

(7) $$e\left(t\right)=\frac{1}{C_{s}\cdot S^{\prime }}\sum_{c=1}^{C_{s}}\sum_{f=1}^{S^{\prime }}F_{c,t,f},\;t=1,\ldots ,T^{\prime }.$$

Next, the time vector $e\in R^{T^{\prime }}$ is fed into a two-layer MLP. After dimensionality reduction via a bottleneck layer, it is mapped back to the original time dimension to obtain the time-step-wise gating weights:

(8) $$g=\sigma \;\left(W_{2}\cdot \mathrm{GELU}\left(W_{1}e+b_{1}\right)+b_{2}\right)\in {\left(0,1\right)}^{T^{\prime }},$$

Here, σ(·) denotes the sigmoid function, which is used to constrain the weights to the interval (0,1). The GELU activation function is used to maintain the smoothness of the nonlinear transformation [29]. Finally, the gated weights are applied element-wise to the feature map via broadcast:

(9) $${\hat{F}}_{c,t,f}=g\left(t\right)\cdot F_{c,t,f}.$$

The proposed temporal gating module does not modify the original temporal order of the sequence but instead redistributes the importance of different temporal positions through continuous weighting. Compared with methods based on predefined fixed windows or manually selected segments, this module does not impose prior assumptions on the location or duration of informative temporal intervals. Instead, it adaptively generates temporal weights according to the CSI response of each input sample. As a result, it is more suitable for handling temporal variations caused by different robotic arm motion speeds and motion patterns.

To avoid excessive disturbance to CSI features that are still unstable during the early stage of training, the parameters of the final layer in the gating predictor are initialized to zero. Under this setting, the weights assigned to different time steps remain approximately uniform at the beginning of training, allowing the model to first learn basic CSI representations before gradually establishing the emphasis and suppression mechanism for key temporal segments. This design helps improve training stability.

**Figure 2:**
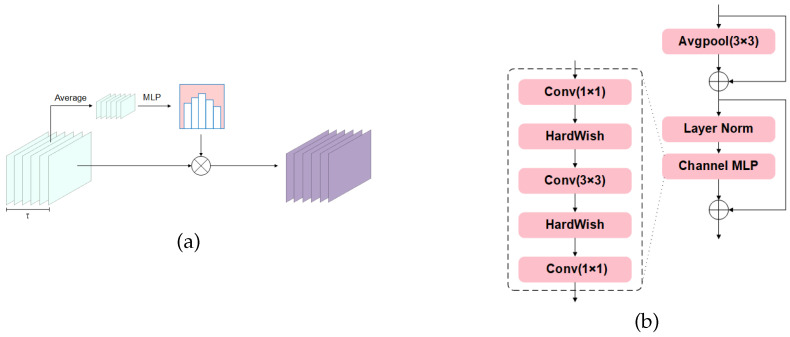
Illustrations of the core modules. (**a**) Temporal gating module. (**b**) Transformer-based feature encoding block with a convolutional channel MLP.

Figure 2a illustrates the overall workflow of the temporal gating module. The features are first compressed along the spatial and frequency dimensions, after which time-step-wise gating weights are predicted and multiplied back into the feature map, thereby explicitly performing temporal reweighting before tokenization.

#### 3.2.4. Patch Embedding

The gated feature map $\hat{F}\in R^{C_{s}\times T^{\prime }\times S^{\prime }}$ is projected onto a sequence of tokens via a convolutional layer with a stride equal to the kernel size. This convolution divides the feature map into several non-overlapping patches and maps each patch to a *D*-dimensional embedding vector, yielding *N* tokens. To preserve the positional information of the patches within the two-dimensional feature map, this paper introduces a learnable positional encoding for each token:

(10) $$Z^{\left(0\right)}=\mathrm{PatchProj}\left(\hat{F}\right)+E_{\mathrm{pos}}\in R^{N\times D}.$$

The features refined by the adaptive temporal downsampling module and temporal gating module are converted into compact token representations through patch embedding and then used as the input to the subsequent Transformer-based feature encoding module.

#### 3.2.5. Transformer-Based Feature Encoding Module

Patch embedding reorganizes the token sequence into a two-dimensional feature map and subsequently feeds it into multiple layers of lightweight encoding modules. The adaptive temporal downsampling module primarily serves to extract local time–frequency responses and reduce redundancy in the frequency dimension, while the temporal gating module further highlights informative temporal intervals. Through these two stages, category-relevant local CSI cues are retained in the form of compact token representations. However, the relationships among these local cues still need to be further integrated so as to form stable, high-level discriminative features.

For robotic arm CSI sensing, inter-class differences are usually not determined by the response of a single local block alone but rather by the joint variation patterns of multiple local subcarrier responses along the temporal dimension. Therefore, the role of the back-end encoder is not to rediscover local perturbations from the raw CSI input but to further integrate the filtered and informative CSI cues within a compact representation space. To meet this requirement, PoolFormer [30] is adopted as the back-end encoding module to perform token interaction and representation refinement with relatively low computational complexity.

First, the compact tokens obtained after patch embedding are reorganized as the input to the back-end encoding stage. Then, contextual information is progressively aggregated among neighboring CSI tokens through pooling operations, rather than through explicit global pairwise interactions. In this way, the remaining informative responses can be further integrated after local enhancement and temporal filtering. Finally, PoolFormer enables efficient high-level aggregation of local CSI cues while maintaining a more favorable balance between recognition performance and computational complexity.

During the spatial mixing phase, this paper employs a neighborhood aggregation method based on local pooling, rather than explicitly computing the pairwise similarities between all tokens. For the input feature map Z, the pooling operations process is represented as

(11) $$Z^{\prime }=Z+\mathrm{DropPath}\;\left(\mathrm{AvgPool}(Z)-Z\right).$$

Here, DropPath denotes stochastic depth regularization [31]. This step facilitates information exchange between adjacent tokens through pooling interactions within local neighborhoods, without the need to explicitly compute global attention. For CSI inputs that have undergone local pattern enhancement and temporal filtering in the earlier stages, this lightweight spatial mixing is sufficient to meet the requirements for subsequent discriminative representation updates.

The model further updates the feature representations through a convolutional channel transformation module:

(12) $$Z^{\left(l+1\right)}=Z^{\prime }+\mathrm{DropPath}\;\left(\mathrm{MLP}(\mathrm{LN}\left(Z^{\prime }\right))\right),$$

where LN denotes layer normalization [32]. Specifically, the MLP consists of three components: point-wise convolution for dimensionality increase, depthwise separable convolution, and point-wise convolution for dimensionality reduction, with the Hardswish activation function used in the middle layer [33]. This architecture preserves a certain degree of local spatial modeling capability while performing channel transformations, thereby helping to further refine the CSI token representations. The detailed structure of a single lightweight Transformer encoding block is illustrated in Figure 2b.

#### 3.2.6. Classification Head

The feature maps produced by the *L* lightweight Transformer encoding blocks are further normalized and aggregated by global average pooling along the spatial dimension, yielding a global feature vector $z\in R^{D}$. Finally, a single layer of normalization and a linear mapping are applied to generate the classification prediction:

(13) $$\hat{y}=\mathrm{Linear}\;\left(\mathrm{LN}\;\left(\mathrm{GAP}\;\left({\mathrm{LN}}_{2d}\left(Z^{\left(L\right)}\right)\right)\right)\right).$$

Here, LN denotes the standard layer normalization applied to vector representations, while ${\mathrm{LN}}_{2d}$ is used for two-dimensional feature maps $X\in R^{B\times C\times H\times W}$. Specifically, ${\mathrm{LN}}_{2d}$ first permutes the feature map to B×H×W×C, applies layer normalization along the channel dimension *C* at each spatial location, and then permutes the result back to B×C×H×W.

## 4. Experimental Setup and Results

### 4.1. Datasets and Evaluation Setup

The proposed method is evaluated on two publicly available robotic arm CSI datasets. Among them, RoboMNIST is used as the primary benchmark for fine-grained motion recognition, while RoboFiSense is employed to further validate the applicability of the proposed method to related robotic arm motion recognition tasks. Table 1 summarizes the main characteristics of the two datasets and the evaluation protocols adopted in this study.

#### 4.1.1. RoboMNIST Dataset

RoboMNIST [22] is a multimodal dataset developed for multi-robot activity recognition. In this dataset, two Franka Emika Panda robotic arms are programmed to write the digits 0–9 on a vertical virtual plane, corresponding to ten activity categories. To simulate motion variability in real-world scenarios, multiplicative uniform noise, ϵ∼U(0.7,1.3), is applied to the first three robot joints during data acquisition, increasing the effective trajectory deviation from the native repeatability of 0.1 mm to approximately 32 cm. Data are collected using three sensing modules, each equipped with a Raspberry Pi 4 running Nexmon firmware [34] as a CSI sniffer under the IEEE 802.11ac standard with an 80 MHz bandwidth. Each recording lasts 15 s with a sampling rate of 30 Hz, and each sensing module produces a complex-valued CSI matrix $H\in C^{450\times 256}$. The dataset covers three motion speed settings, namely, high, medium, and low, and each combination of activity category, robot, and speed contains at least 32 repetitions. In this study, the CSI amplitude data from two sniffers are concatenated along the channel dimension and used as the model input, resulting in a total of 2135 samples for classification.

#### 4.1.2. RoboFiSense Dataset

RoboFiSense [21] is the first publicly available CSI benchmark for robotic arm motion recognition. In this dataset, a Franka Emika robotic arm performs eight action categories, namely, Arc, Elbow, Rectangle, Silence, SLFW, SLRL, SLUD, and Triangle. Meanwhile, two Raspberry Pi-based sniffers synchronously collect CSI data at a sampling rate of 30 Hz within a 12 s acquisition window, producing $H\in C^{360\times 256}$. After removing pilot subcarriers, guard bands, and the DC subcarrier, 232 valid subcarriers are retained. The dataset was collected under four spatial sniffer configurations and three robotic arm speed settings in order to investigate the sensitivity of Wi-Fi sensing to changes in environmental and operational conditions. In this study, the CSI amplitude data from the two sniffers are fused and a total of 552 samples are used to evaluate the applicability of the proposed method to robotic arm motion recognition tasks.

### 4.2. Training Configuration

The model was trained using the AdamW optimizer [35] with a cosine-annealing learning rate schedule and a linear warm-up strategy [36]. The loss function was set to cross-entropy. Gradient clipping was applied during training to improve optimization stability and validation accuracy was used as the criterion for early stopping to reduce the risk of overfitting. Except for the cross-speed evaluation on RoboFiSense, all experiments were conducted using five-fold stratified cross-validation and the mean and standard deviation were reported. In addition, all cross-validation experiments were performed under a fixed random seed to reduce the influence of data partition variability. Table 2 lists the training hyperparameter settings for the two datasets.

Table 3 summarizes the key architecture hyperparameters of MSPoolNet used in the experiments.

### 4.3. Experimental Environment

All experiments were conducted on a workstation equipped with an NVIDIA GeForce RTX 5090 GPU using Python 3.12 and PyTorch 2.7.0. The evaluation metrics included test accuracy, macro-average F1 score (Macro-F1), macro-average precision (Macro-Precision), macro-average recall (Macro-Recall), and single-sample inference latency. The reported latency refers to the model inference latency, i.e., the average forward-pass time of the recognition model after the CSI tensor is constructed. It does not include CSI acquisition, window buffering, amplitude extraction, normalization, tensor construction, or data-transfer time. Specifically, letting *C* denote the number of classes and ${\mathrm{TP}}_{c}$, ${\mathrm{FP}}_{c}$, and ${\mathrm{FN}}_{c}$ denote the true positives, false positives, and false negatives for class *c*, with $N=\sum_{c=1}^{C}\left({\mathrm{TP}}_{c}+{\mathrm{FN}}_{c}\right)$ denoting the total number of test samples, these metrics are defined as follows:

(14) $$\mathrm{Accuracy}=\frac{\sum_{c=1}^{C}{\mathrm{TP}}_{c}}{N},$$

(15) $$\mathrm{Macro}\text{-}\mathrm{Precision}=\frac{1}{C}\sum_{c=1}^{C}\frac{{\mathrm{TP}}_{c}}{{\mathrm{TP}}_{c}+{\mathrm{FP}}_{c}},$$

(16) $$\mathrm{Macro}\text{-}\mathrm{Recall}=\frac{1}{C}\sum_{c=1}^{C}\frac{{\mathrm{TP}}_{c}}{{\mathrm{TP}}_{c}+{\mathrm{FN}}_{c}},$$

(17) $$\mathrm{Macro}\text{-}F1=\frac{1}{C}\sum_{c=1}^{C}\frac{2\;{\mathrm{TP}}_{c}}{2\;{\mathrm{TP}}_{c}+{\mathrm{FP}}_{c}+{\mathrm{FN}}_{c}}.$$

Standard experiments were performed using five-fold stratified cross-validation, with the mean and standard deviation reported. For the cross-speed experiments, a leave-one-speed-out evaluation protocol was adopted, and the mean and standard deviation across the three speed partitions were reported. To ensure comparability, all baseline models implemented in this study followed the same data preprocessing pipeline, early stopping strategy, and training protocol as the proposed method. In particular, the RoboFiSense baseline comparisons were obtained by re-training all baseline models under the same experimental configuration as MSPoolNet, including the same data split protocol, preprocessing pipeline, training strategy, early stopping criterion, and evaluation metrics.

### 4.4. Baseline Methods

To evaluate the overall recognition performance of MSPoolNet, we compare it with several representative baseline models covering convolutional, recurrent, hybrid spatiotemporal, Transformer-based, and recent task-related architectures.

CNN is adopted as a representative convolutional baseline for modeling local patterns in CSI representations. It mainly reflects the capability of convolutional architectures for extracting local time–frequency features.

LSTM is adopted as a recurrent baseline that treats the CSI amplitude matrix as a temporal sequence, where the subcarrier vector at each time step is regarded as the input vector and the final hidden state is used for classification.

BiLSTM extends the above recurrent formulation by modeling temporal dependencies in both forward and backward directions. The hidden states from the two directions are concatenated before classification.

ConvLSTM [37] is adopted as a hybrid spatiotemporal baseline. By replacing the standard matrix multiplications in the gating operations with convolutions, it preserves the two-dimensional CSI structure during temporal modeling.

Transformer is adopted as a standard Transformer encoder baseline. The CSI matrix is partitioned into fixed-length temporal chunks as input tokens and mean pooling over the output sequence is used for classification.

ViT is adopted as a standard Vision Transformer baseline. The two-dimensional CSI amplitude map is divided into non-overlapping patch tokens and encoded by stacked Transformer blocks, and the final aggregated representation is used for classification.

BiVTC is included as a task-related Transformer baseline for robotic arm WiFi sensing. It adopts a dual-branch ViT architecture to process two independent sniffer inputs in parallel and concatenates the corresponding token representations before classification.

RoboDSTAN is included as a recent task-related baseline for robotic arm motion recognition from WiFi sensing. It adopts a dual-stream temporal convolutional architecture with self-attention, fuses the two sniffer streams through concatenation, and performs final prediction with an MLP classifier.

### 4.5. Results on RoboMNIST

To evaluate the overall recognition performance of MSPoolNet on fine-grained robotic arm motion recognition, we compare it with several representative baseline models on the RoboMNIST dataset.

Table 4 summarizes the five-fold cross-validation results of different models on the RoboMNIST dataset. In terms of absolute classification performance, the standard Transformer with a relatively larger parameter scale achieves the highest accuracy and Macro-F1 score, indicating that global token interaction provides strong representation capability for this fine-grained motion recognition task. However, this model contains 1.9 M parameters and exhibits substantially higher inference latency than the proposed method, suggesting that its performance advantage is achieved at the cost of increased model complexity.

By comparison, the proposed method attains 99.44% accuracy and 99.44% Macro-F1 with only 368 K parameters, achieving the best performance among all lightweight models and demonstrating a more favorable balance among parameter efficiency, inference latency, and classification accuracy. Compared with ConvLSTM, CNN, RoboDSTAN, LSTM, and BiLSTM, which have comparable parameter scales, the proposed method consistently achieves better classification performance. These results indicate that the combined design of an adaptive temporal downsampling module, temporal gating module, and efficient pooling operations is more suitable for fine-grained robotic arm motion recognition from CSI than architectures relying only on recurrent modeling or convolutional stacking.

In addition, the proposed method and the standard Transformer both clearly outperform ViT and BiVTC on this dataset. This result suggests that directly transferring a visual Transformer architecture to the RoboMNIST task does not necessarily lead to satisfactory performance. Instead, the design of the adaptive temporal downsampling module and the choice of lightweight pooling operations remain critical for effective CSI-based fine-grained motion recognition.

**Figure 3:**
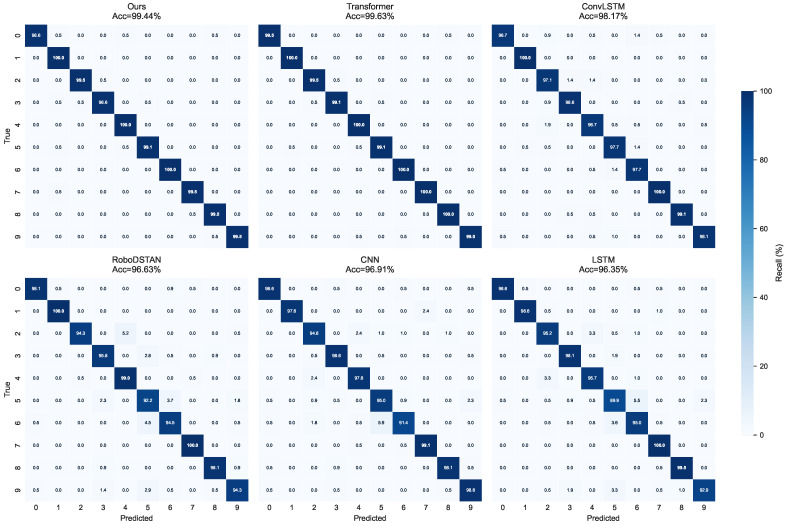
Comparison of confusion matrices on the RoboMNIST dataset (aggregated over five-fold cross-validation and row-normalized to recall percentages). The subplot titles report the average accuracy of each model.

To further evaluate the class-level discriminative capability of the proposed method, Figure 3 shows the confusion matrices of the proposed method and several representative baseline models on the RoboMNIST dataset. Both the proposed method and the standard Transformer exhibit highly concentrated diagonal responses, whereas ConvLSTM, RoboDSTAN, CNN, and LSTM show more evident off-diagonal confusion in several categories. These results indicate that the proposed method can distinguish fine-grained motion categories more reliably while maintaining relatively low model complexity, and that its class-level discriminative capability is overall superior to that of most lightweight baseline models.

To further evaluate the efficiency of the proposed method, Table 5 compares several representative models in terms of parameter count, classification accuracy, and single-sample inference latency. Under comparable parameter scales, the proposed method achieves the highest classification accuracy among ConvLSTM, CNN, RoboDSTAN, and LSTM. Compared with the standard Transformer, the proposed method shows only a 0.19 percentage point decrease in accuracy, while reducing the parameter count and inference latency to approximately one-fifth and three-fifths of those of the standard Transformer, respectively. These results indicate that the proposed method provides a more favorable trade-off between efficiency and performance.

In addition, the proposed method achieves 2.53 percentage points higher accuracy than CNN, despite CNN exhibiting slightly lower inference latency. Compared with ConvLSTM, the proposed method achieves higher accuracy while maintaining substantially lower inference latency (0.65 ms vs. 3.65 ms). Overall, the proposed method delivers a more balanced performance under lightweight deployment constraints.

**Figure 4:**
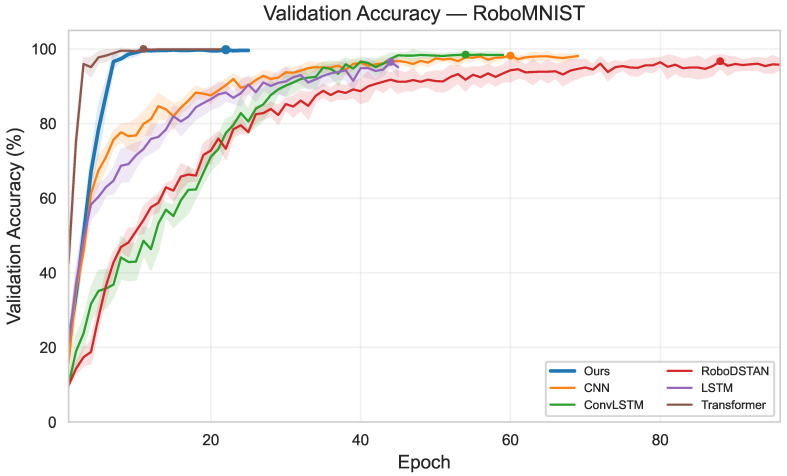
Validation accuracy curves of the main models over training epochs on the RoboMNIST dataset (five-fold mean ± standard deviation).

Figure 4 illustrates the training dynamics of the major models. As shown, the proposed method reaches a high validation accuracy at an early stage of training and exhibits only minor fluctuations across subsequent epochs, indicating fast convergence and stable generalization performance on the current task.

### 4.6. Results on RoboFiSense

To further examine the applicability of MSPoolNet beyond the primary fine-grained benchmark, we conduct comparative experiments on RoboFiSense, which corresponds to a related robotic arm activity recognition task.

Table 6 presents the five-fold cross-validation results on the RoboFiSense dataset. To avoid protocol inconsistency in the RoboFiSense comparison, all baseline models were re-trained and evaluated under the same five-fold protocol using the same data split strategy. On this validation task, the proposed method achieves the best overall performance, reaching 99.46% in both accuracy and Macro-F1 while maintaining low model inference latency. Compared with the baseline models, the proposed method shows consistent improvements in accuracy, F1 score, precision, and recall, indicating that the proposed architecture is not only effective for fine-grained motion recognition on RoboMNIST but also transferable to related robotic arm motion recognition tasks.

These results further suggest that the adaptive temporal downsampling module for local spatiotemporal representation learning, the temporal gating module for informative segment selection, and the efficient pooling operations of the Transformer-based feature encoding module exhibit good generalization ability across different robotic arm CSI classification tasks. From the perspective of task characteristics, the class differences in RoboFiSense are more related to coarse-grained motion patterns than those in RoboMNIST, which makes this dataset comparatively easier to distinguish and leads to higher and more stable overall performance.

The revised results show that MSPoolNet improves the accuracy by 2.27 percentage points compared with RoboDSTAN and reduces model inference latency from 5.65 ms to 1.29 ms. This result demonstrates that the collaborative design of the adaptive temporal downsampling module, temporal gating module, and Transformer-based feature encoding module is effective for the current task and indicates that an architecture tailored to CSI characteristics does not necessarily sacrifice recognition accuracy.

It is worth noting that the proposed method achieves better recognition performance with a lower parameter scale than BiVTC, which adopts a dual-branch ViT architecture with a relatively larger parameter scale. This characteristic makes the proposed architecture more suitable for deployment in resource-constrained scenarios.

### 4.7. Cross-Speed Generalization on RoboFiSense

To assess the robustness of the proposed method under motion-speed variation, we further conduct leave-one-speed-out experiments on RoboFiSense.

Table 7 presents the cross-speed evaluation results on the RoboFiSense dataset. To further examine motion-speed generalization under a consistent comparison protocol, we re-evaluated all baselines under the same leave-one-speed-out setting on RoboFiSense. For each fold, two speeds were used for training and the remaining speed was used for testing. Across the three leave-one-speed-out partitions, the proposed method achieves an average accuracy of 99.09%, outperforming all baseline models, and demonstrates strong adaptability under unseen speed conditions. Table 7 reports the class-level recall for each held-out speed partition, with the corresponding cross-partition standard deviation computed across the three speed partitions for each model and class. At the class level, the proposed method maintains high recall for most categories across different speed partitions. Only the Rectangle category shows a larger standard deviation, which indicates that this class is more sensitive to speed-induced temporal distribution shifts. This result suggests that the temporal distribution shifts introduced by speed variation do not affect all classes equally and that categories whose trajectory patterns are more dependent on execution rhythm are more sensitive to changes in motion speed.

Overall, the cross-speed experiments show that the proposed method is effective not only under standard in-distribution classification settings but also under speed-shifted conditions. At the same time, these results indicate that robustness to motion speed variation remains an important issue for further study in robotic arm Wi-Fi sensing. Compared with RoboDSTAN, MSPoolNet achieves a higher mean overall recall with lower variation across speed partitions (99.09 ± 1.57% vs. 95.47 ± 2.26%). This comparison indicates that the proposed method is not only effective under conventional five-fold cross-validation but also exhibits stronger stability under unseen speed conditions.

From a methodological perspective, this improvement may be attributed to the robust extraction of local spatiotemporal patterns by the adaptive temporal downsampling module and the adaptive emphasis on informative motion segments introduced by the temporal gating module, which together enable the model to preserve strong discriminative capability under temporal stretching or compression caused by speed variation.

### 4.8. Test-Time Signal Degradation Analysis

To further verify the robustness of MSPoolNet from the perspective of signal perturbation, we conduct a test-time signal degradation experiment on both RoboMNIST and RoboFiSense. In this experiment, the model is trained using the original clean training samples, while artificial degradation is applied only to the test samples for inference. Four representative degradation settings are considered: Gaussian noise, random subcarrier dropout, contiguous frequency masking, and temporal shifting. These settings simulate different forms of CSI corruption, including measurement noise, partial subcarrier loss, localized frequency-band disturbance, and temporal misalignment.

As shown in Table 8, MSPoolNet maintains high recognition accuracy under mild signal degradation, while stronger degradation produces more visible performance decreases. On RoboMNIST, Gaussian noise has little influence across σ=0.05 to σ=0.15, with the accuracy remaining at 99.44% and the decrease limited to 0.04 percentage points. Under subcarrier dropout, the accuracy remains at 99.39% at 2% dropout and 98.50% at 5% dropout, but decreases more noticeably to 95.78% when the dropout ratio increases to 10%. For contiguous frequency masking, the accuracy decreases from 98.92% with C subcarriers to 97.19% and 95.18% with 16 and 24 masked subcarriers, respectively. Under temporal shifting, the model achieves 99.06%, 97.94%, and 95.22% accuracy for shifts of 10, 20, and 30 samples, respectively. These results indicate that removing or misaligning a larger portion of CSI components can cause an evident performance decline. The larger decreases caused by subcarrier dropout and frequency masking can be attributed to their direct disruption of frequency-domain CSI structures.

On RoboFiSense, the influence of these perturbations is generally smaller under the same selected levels. The accuracy remains 99.73% under Gaussian noise with σ=0.05 and σ=0.10, and only decreases by 0.09 percentage points when σ=0.15. Under subcarrier dropout, the accuracy remains at 99.37% and 99.46% at dropout ratios of 2% and 5%, respectively, and decreases to 98.09% at 10% dropout. For frequency masking, the accuracy decreases from 99.55% with 8 masked subcarriers to 99.00% and 98.01% with 16 and 24 masked subcarriers, respectively. Under temporal shifts of 10, 20, and 30 samples, the corresponding accuracies are 99.73%, 99.64%, and 99.28%. These results indicate, from the perspective of artificial signal degradation, that the proposed architecture retains stable recognition performance when representative signal components are mildly perturbed or partially missing, while also showing that stronger subcarrier loss, frequency masking, and temporal misalignment can cause clear performance decreases. However, these results should be interpreted as evidence of robustness under selected synthetic test-time signal degradation levels, and they do not fully demonstrate robustness under real deployment conditions such as environmental changes, device repositioning, hardware variation, or real Wi-Fi interference.

### 4.9. Ablation Study

To quantify the contribution of each key module and structural design choice, ablation experiments are conducted on RoboMNIST.

**Figure 5:**
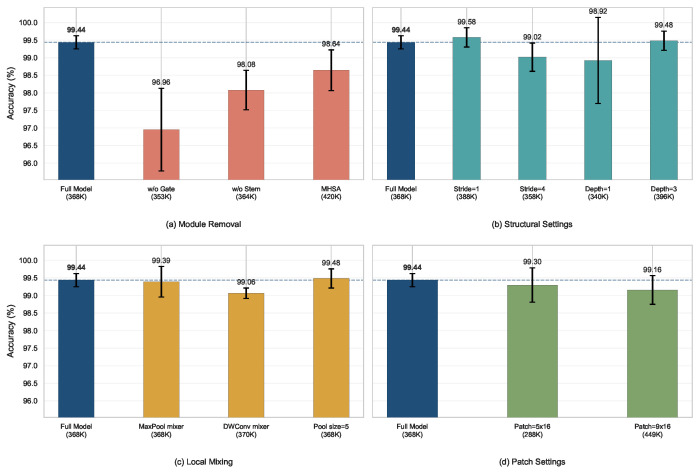
Ablation study results on the RoboMNIST dataset. (**a**) Module removal variants. (**b**) Structural setting variants. (**c**) Local mixing variants. (**d**) Patch setting variants. Each subplot reports the five-fold mean ± standard deviation and the dashed line marks the default configuration.

Figure 5 summarizes the ablation results on the RoboMNIST dataset. Overall, the results indicate that the performance gain of the proposed method does not come from any single component. Instead, it arises from the joint effect of the adaptive temporal downsampling module, temporal gating module, and Transformer-based feature encoding module.

As shown in Figure 5a, removing the temporal gating module causes the largest accuracy drop, indicating that explicitly emphasizing informative temporal segments within a fixed-length CSI window is crucial for the target task. Removing the adaptive temporal downsampling module also leads to a clear performance degradation, which suggests that the subsequent tokenization and encoding stages alone are insufficient to fully characterize the subtle local responses contained in robotic arm CSI signals. When the lightweight Transformer encoding blocks are replaced by standard self-attention, the accuracy decreases slightly, while the model size and inference cost increase. This result suggests that lightweight pooling operations are more suitable than standard attention for achieving a favorable balance between recognition performance and computational complexity in this setting.

Figure 5b presents the influence of different structural settings. Although Stride =1 yields a slightly higher accuracy than the default setting, it also increases the parameter count and computational cost, indicating that a higher input resolution does not substantially improve the overall performance ceiling. Depth =1 reduces latency further, but the corresponding accuracy drop is more pronounced. By contrast, Depth =3 provides only limited improvement, suggesting that simply increasing the number of back-end encoding layers is not the most effective way to improve performance on the current task.

Figure 5c,d further compare the effects of local mixing settings and patch settings. The results show that different back-end configurations have varying influences on both performance and efficiency. In particular, a larger local mixing kernel brings a slight accuracy gain, but it also increases inference latency. Meanwhile, neither finer nor coarser patch partitioning consistently outperforms the default configuration.

Overall, although the default setting is not optimal for every individual metric, it provides a more reasonable overall trade-off among accuracy, stability, model size, and inference latency.

**Figure 6:**
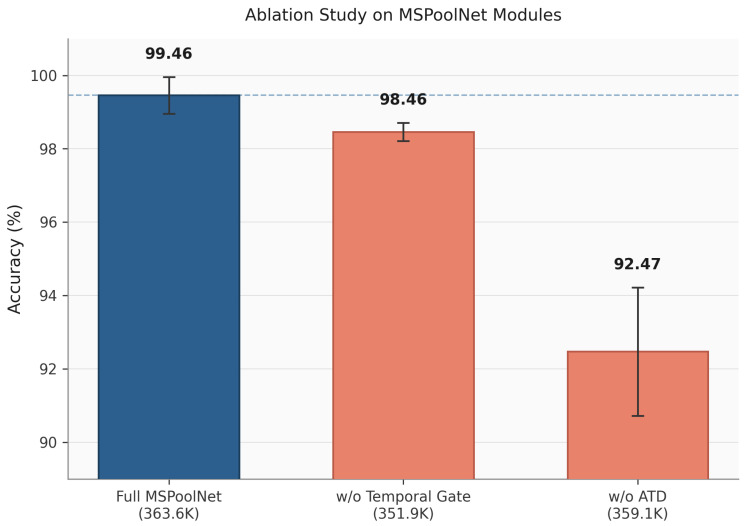
Key ablation results on RoboFiSense. The bars report five-fold mean accuracy with standard deviation.

To further examine whether the key components remain effective on RoboFiSense, we additionally evaluated two representative ablated variants under the same five-fold protocol. As shown in Figure 6, the full MSPoolNet achieves 99.46% accuracy on RoboFiSense. Removing the temporal gate decreases the accuracy to 98.46%, corresponding to a 1.00 percentage point drop. This result indicates that adaptive temporal weighting also contributes to RoboFiSense, although the effect is relatively moderate on this dataset. By contrast, replacing the adaptive temporal downsampling/convolutional stem leads to a larger decrease, with accuracy dropping to 92.47%. This suggests that the front-end local time–frequency feature extraction and downsampling module is a more critical component for preserving discriminative CSI patterns before tokenization. Overall, the additional RoboFiSense ablation confirms that the two main temporal modules are not only effective on RoboMNIST but also contribute under the RoboFiSense recognition setting.

### 4.10. Phase Feature Comparison

To further examine whether the CSI phase provides additional recognition information, we conducted a brief phase feature comparison experiment on RoboMNIST. The same MSPoolNet architecture and five-fold evaluation protocol were used, while only the input feature setting was changed. Four input settings were compared. The amplitude-only result in this comparison is reported as the internal reference under the same experimental configuration as the phase feature comparison, rather than as a replacement for the main RoboMNIST result in Table 4. The amplitude-only setting uses the CSI magnitude as the input. The sanitized phase-only setting uses phase features after temporal unwrapping and packet-wise linear trend removal along subcarriers. The two amplitude–phase settings concatenate the amplitude feature with either temporally unwrapped phase or sanitized phase.

As shown in Table 9, the sanitized phase-only setting performs poorly, while adding phase to amplitude leads to only a marginal change compared with the amplitude-only setting. These results indicate that phase information does not provide a practically meaningful improvement over the amplitude-only setting in this experiment. Therefore, the current study keeps MSPoolNet as an amplitude-based lightweight model, while reliable phase-amplitude fusion remains a future direction requiring more careful calibration and robustness evaluation.

## 5. Conclusions

This study investigates fine-grained robotic arm motion recognition from CSI amplitude for contactless robotic state sensing. To accommodate weak local CSI perturbations, temporally sparse discriminative cues, and lightweight deployment requirements, MSPoolNet is developed by integrating an adaptive temporal downsampling module, a temporal gating module, and a lightweight pooling-based Transformer encoding module. Through this design, the model enhances local time–frequency responses, emphasizes informative temporal segments, and aggregates compact token representations for final recognition.

Experimental results on RoboMNIST and RoboFiSense demonstrate the effectiveness of the proposed method for robotic arm motion recognition. In particular, MSPoolNet achieves strong recognition performance while maintaining a compact model structure. The cross-speed, ablation, and test-time signal degradation results further indicate that the proposed architecture provides a favorable balance among recognition accuracy, model efficiency, and robustness.

Nevertheless, the robustness evaluation in the current study is still limited. Although test-time signal degradation experiments are conducted to examine the influence of noise, subcarrier loss, frequency masking, and temporal misalignment, these experiments do not fully represent real-environment robustness. In particular, the current study does not yet include systematic evaluations under room layout changes, transmitter/receiver placement variations, realistic Wi-Fi interference, hardware differences, or long-term environmental dynamics. Such deployment-oriented robustness requires more comprehensive data collection and evaluation protocols across diverse environments, positions, and interference conditions.

Another limitation is that this study mainly focuses on CSI amplitude. Although the phase feature comparison indicates that adding phase may provide a slight accuracy improvement, the observed gain is marginal under the current setting. Considering the trade-off between the additional calibration and fusion complexity introduced by phase processing and the limited performance benefit observed in this experiment, we did not further develop a phase-aware model in the present study. Future work can therefore investigate more reliable phase calibration and phase-guided model design, especially under speed variation, signal degradation, and real deployment changes.

Future work will therefore focus on more comprehensive, real-world robustness evaluation, online deployment validation (e.g., on embedded platforms such as Raspberry Pi), and reliable phase-aware or phase-amplitude fusion models to further improve the practicality of CSI-based robotic arm sensing under complex deployment conditions.

## Figures and Tables

**Table 1:** Summary of the two CSI datasets used in this study.

Property	RoboMNIST	RoboFiSense
Number of classes	10	8
Total samples	2135	552
Time steps (*T*)	450	360
Subcarriers (*S*)	256	232
Receivers (sniffers)	3	2

**Table 2:** Training hyperparameters used in this study.

Hyperparameter	RoboMNIST	RoboFiSense
Optimizer	AdamW	AdamW
Learning rate	$1\times 10^{-3}$	$1\times 10^{-3}$
Weight decay	$1\times 10^{-2}$	$1\times 10^{-2}$
Batch size	64	64
Epochs	150	150
Warmup epochs	3	3
Scheduler	Cosine	Cosine
Gradient clipping	1.0	1.0
Early stopping	patience = 15	patience = 15

**Table 3:** Key architecture hyperparameters of MSPoolNet used in the experiments.

Module	Hyperparameter	Value
ATD/stem	Convolutional layers	2
	Kernel size	3×3
	Output channels	16,32
Temporal gate	Bottleneck dimension	32
Patch embedding	Patch size	7×16
	Embedding dimension	80
Encoder	Encoder blocks	2
	Token-mixer kernel	3×3
	Channel-MLP expansion	2.0

**Table 4:** Five-fold cross-validation results on the RoboMNIST dataset. Values are reported as five-fold mean ± standard deviation.

Model	Params	Acc. (%)	F1 (%)	Prec. (%)	Recall (%)	Lat. (ms)
Ours	368 K	99.44 ± 0.19	99.44 ± 0.19	99.44 ± 0.19	99.44 ± 0.19	0.65 ± 0.02
Transformer	1.9 M	99.63 ± 0.38	99.63 ± 0.38	99.63 ± 0.37	99.63 ± 0.38	1.09 ± 0.19
ConvLSTM	372 K	98.17 ± 0.52	98.17 ± 0.53	98.21 ± 0.51	98.17 ± 0.52	3.65 ± 0.11
CNN	369 K	96.91 ± 0.99	96.92 ± 1.01	97.01 ± 0.95	96.93 ± 1.01	0.52 ± 0.01
RoboDSTAN	361 K	96.63 ± 1.03	96.65 ± 1.01	96.76 ± 0.96	96.65 ± 1.03	1.64 ± 0.22
LSTM	371 K	96.35 ± 1.45	96.35 ± 1.45	96.44 ± 1.42	96.35 ± 1.42	2.11 ± 0.03
BiLSTM	371 K	92.60 ± 2.29	92.58 ± 2.31	92.79 ± 2.22	92.61 ± 2.31	0.71 ± 0.07
BiVTC	2.8 M	66.98 ± 3.22	66.75 ± 3.61	69.02 ± 3.99	67.07 ± 3.17	2.49 ± 0.17
ViT	1.4 M	61.45 ± 4.02	61.33 ± 3.89	62.52 ± 3.40	61.56 ± 4.05	2.00 ± 0.10

**Table 5:** Efficiency comparison of representative models on the RoboMNIST dataset. Accuracy and latency are reported as five-fold mean ± standard deviation.

Model	Params	Acc. (%)	Lat. (ms)
**Ours**	368 K	99.44 ± 0.19	0.65 ± 0.02
Transformer	1.9 M	99.63 ± 0.38	1.09 ± 0.19
ConvLSTM	372 K	98.17 ± 0.52	3.65 ± 0.11
CNN	369 K	96.91 ± 0.99	0.52 ± 0.01
RoboDSTAN	361 K	96.63 ± 1.03	1.64 ± 0.22
LSTM	371 K	96.35 ± 1.45	2.11 ± 0.03

**Table 6:** Five-fold cross-validation results on the RoboFiSense dataset. All baseline models were re-trained under the same protocol.

Model	Params	Acc. (%)	F1 (%)	Prec. (%)	Recall (%)	Lat. (ms)
MSPoolNet	363.6 K	99.46 ± 0.50	99.46 ± 0.50	99.47 ± 0.48	99.45 ± 0.50	1.29 ± 0.03
RoboDSTAN	354.5 K	97.19 ± 1.13	97.21 ± 1.14	97.42 ± 0.96	97.18 ± 1.16	5.65 ± 0.10
BiVTC	2.76 M	55.22 ± 5.16	54.77 ± 4.74	56.96 ± 5.30	55.16 ± 5.06	2.44 ± 0.06
ViT	1.37 M	64.28 ± 4.44	63.98 ± 4.31	66.50 ± 3.05	64.27 ± 4.49	1.88 ± 0.13
BiLSTM	340.7 K	82.23 ± 0.78	82.00 ± 0.84	82.82 ± 0.51	82.20 ± 0.79	0.65 ± 0.01
LSTM	343.5 K	87.22 ± 1.55	87.18 ± 1.50	87.59 ± 1.41	87.19 ± 1.56	1.72 ± 0.05
CNN	361.4 K	85.86 ± 7.35	85.93 ± 7.36	86.79 ± 7.01	85.84 ± 7.38	0.54 ± 0.09
ConvLSTM	372.1 K	90.48 ± 3.98	90.51 ± 3.91	90.87 ± 3.68	90.46 ± 3.95	3.81 ± 0.31

**Table 7:** Per-class recall (%) for leave-one-speed-out evaluation on the RoboFiSense dataset. The value before ± is the recall for the corresponding held-out speed partition. The ±value denotes the cross-partition standard deviation computed across the three speed partitions for the same model and class; therefore, it is repeated across the three rows of the same model for the same class.

Train	Test	Model	Arc	Elbow	Rect.	Silence	SLFW	SLRL	SLUD	Tri.	Overall
V1 & V2	V3	MSPoolNet	100.00 ± 0.00	100.00 ± 0.00	100.00 ± 12.55	100.00 ± 0.00	100.00 ± 0.00	100.00 ± 0.00	100.00 ± 0.00	100.00 ± 0.00	100.00 ± 1.57
		RoboDSTAN	95.65 ± 10.04	95.65 ± 2.51	95.65 ± 10.94	86.96 ± 4.35	100.00 ± 0.00	95.65 ± 2.51	100.00 ± 0.00	100.00 ± 0.00	96.20 ± 2.26
		BiVTC	34.78 ± 6.64	73.91 ± 30.43	69.57 ± 4.35	47.83 ± 18.10	52.17 ± 20.55	17.39 ± 33.77	82.61 ± 23.95	65.22 ± 23.01	55.43 ± 9.03
		BiLSTM	69.57 ± 13.97	100.00 ± 4.35	100.00 ± 13.28	52.17 ± 8.70	86.96 ± 7.53	65.22 ± 15.06	91.30 ± 7.53	100.00 ± 17.57	83.15 ± 4.01
		LSTM	52.17 ± 21.88	95.65 ± 2.51	95.65 ± 0.00	56.52 ± 12.55	95.65 ± 2.51	65.22 ± 13.97	100.00 ± 8.70	91.30 ± 2.51	81.52 ± 4.36
		ViT	52.17 ± 27.61	78.26 ± 15.67	82.61 ± 41.93	43.48 ± 15.27	73.91 ± 26.09	60.87 ± 13.28	73.91 ± 21.45	95.65 ± 34.51	70.11 ± 4.56
		CNN	8.70 ± 45.25	95.65 ± 25.47	91.30 ± 10.04	86.96 ± 6.64	73.91 ± 2.51	95.65 ± 15.27	100.00 ± 4.35	47.83 ± 11.51	75.00 ± 2.06
V1 & V3	V2	MSPoolNet	100.00 ± 0.00	100.00 ± 0.00	100.00 ± 12.55	100.00 ± 0.00	100.00 ± 0.00	100.00 ± 0.00	100.00 ± 0.00	100.00 ± 0.00	100.00 ± 1.57
		RoboDSTAN	95.65 ± 10.04	100.00 ± 2.51	86.96 ± 10.94	95.65 ± 4.35	100.00 ± 0.00	100.00 ± 2.51	100.00 ± 0.00	100.00 ± 0.00	97.28 ± 2.26
		BiVTC	39.13 ± 6.64	17.39 ± 30.43	65.22 ± 4.35	21.74 ± 18.10	13.04 ± 20.55	82.61 ± 33.77	39.13 ± 23.95	21.74 ± 23.01	37.50 ± 9.03
		BiLSTM	95.65 ± 13.97	95.65 ± 4.35	91.30 ± 13.28	69.57 ± 8.70	100.00 ± 7.53	91.30 ± 15.06	91.30 ± 7.53	78.26 ± 17.57	89.13 ± 4.01
		LSTM	95.65 ± 21.88	91.30 ± 2.51	95.65 ± 0.00	78.26 ± 12.55	100.00 ± 2.51	86.96 ± 13.97	82.61 ± 8.70	91.30 ± 2.51	90.22 ± 4.36
		ViT	100.00 ± 27.61	56.52 ± 15.67	95.65 ± 41.93	26.09 ± 15.27	47.83 ± 26.09	86.96 ± 13.28	39.13 ± 21.45	43.48 ± 34.51	61.96 ± 4.56
		CNN	73.91 ± 45.25	86.96 ± 25.47	91.30 ± 10.04	95.65 ± 6.64	69.57 ± 2.51	82.61 ± 15.27	95.65 ± 4.35	30.43 ± 11.51	78.26 ± 2.06
V2 & V3	V1	MSPoolNet	100.00 ± 0.00	100.00 ± 0.00	78.26 ± 12.55	100.00 ± 0.00	100.00 ± 0.00	100.00 ± 0.00	100.00 ± 0.00	100.00 ± 0.00	97.28 ± 1.57
		RoboDSTAN	78.26 ± 10.04	100.00 ± 2.51	73.91 ± 10.94	91.30 ± 4.35	100.00 ± 0.00	100.00 ± 2.51	100.00 ± 0.00	100.00 ± 0.00	92.93 ± 2.26
		BiVTC	26.09 ± 6.64	26.09 ± 30.43	60.87 ± 4.35	56.52 ± 18.10	43.48 ± 20.55	65.22 ± 33.77	78.26 ± 23.95	30.43 ± 23.01	48.37 ± 9.03
		BiLSTM	91.30 ± 13.97	91.30 ± 4.35	73.91 ± 13.28	60.87 ± 8.70	100.00 ± 7.53	91.30 ± 15.06	78.26 ± 7.53	65.22 ± 17.57	81.52 ± 4.01
		LSTM	78.26 ± 21.88	91.30 ± 2.51	95.65 ± 0.00	56.52 ± 12.55	100.00 ± 2.51	91.30 ± 13.97	91.30 ± 8.70	86.96 ± 2.51	86.41 ± 4.36
		ViT	100.00 ± 27.61	47.83 ± 15.67	17.39 ± 41.93	56.52 ± 15.27	100.00 ± 26.09	69.57 ± 13.28	78.26 ± 21.45	30.43 ± 34.51	62.50 ± 4.56
		CNN	95.65 ± 45.25	47.83 ± 25.47	73.91 ± 10.04	100.00 ± 6.64	69.57 ± 2.51	65.22 ± 15.27	91.30 ± 4.35	52.17 ± 11.51	74.46 ± 2.06

**Table 8:** Quantitative results of MSPoolNet under multiple test-time signal degradation levels on RoboMNIST and RoboFiSense. Values are reported as five-fold mean ± standard deviation.

Dataset	Degradation	Level	Acc. (%)	F1 (%)	Acc. Drop
RoboMNIST	Clean	–	99.48 ± 0.20	99.48 ± 0.19	0.00
	Gaussian noise	σ=0.05	99.44 ± 0.27	99.43 ± 0.27	−0.04
		σ=0.10	99.44 ± 0.21	99.44 ± 0.21	−0.04
		σ=0.15	99.44 ± 0.21	99.44 ± 0.21	−0.04
	Subcarrier dropout	2%	99.39 ± 0.43	99.39 ± 0.42	−0.09
		5%	98.50 ± 0.80	98.50 ± 0.80	−0.98
		10%	95.78 ± 1.21	95.76 ± 1.22	−3.70
	Frequency mask	8	98.92 ± 0.27	98.92 ± 0.26	−0.56
		16	97.19 ± 0.72	97.19 ± 0.72	−2.29
		24	95.18 ± 1.56	95.18 ± 1.56	−4.30
	Temporal shift	10	99.06 ± 0.17	99.06 ± 0.17	−0.42
		20	97.94 ± 0.45	97.94 ± 0.45	−1.54
		30	95.22 ± 1.25	95.22 ± 1.25	−4.26
RoboFiSense	Clean	–	99.73 ± 0.41	99.73 ± 0.41	0.00
	Gaussian noise	σ=0.05	99.73 ± 0.41	99.73 ± 0.41	0.00
		σ=0.10	99.73 ± 0.41	99.73 ± 0.41	0.00
		σ=0.15	99.64 ± 0.38	99.64 ± 0.38	−0.09
	Subcarrier dropout	2%	99.37 ± 0.52	99.37 ± 0.51	−0.36
		5%	99.46 ± 0.38	99.46 ± 0.38	−0.27
		10%	98.09 ± 1.49	98.10 ± 1.49	−1.64
	Frequency mask	8	99.55 ± 0.45	99.55 ± 0.45	−0.18
		16	99.00 ± 0.67	99.00 ± 0.67	−0.73
		24	98.01 ± 0.61	98.01 ± 0.61	−1.72
	Temporal shift	10	99.73 ± 0.41	99.73 ± 0.41	0.00
		20	99.64 ± 0.38	99.64 ± 0.38	−0.09
		30	99.28 ± 0.52	99.28 ± 0.52	−0.45

**Table 9:** Phase feature comparison on RoboMNIST using MSPoolNet.

Input Feature	Phase Processing	Acc. (%)
Amplitude only	–	99.81 ± 0.20
Sanitized phase only	Unwrapping + linear trend removal	12.37 ± 2.08
Amplitude + unwrapped phase	Unwrapping	99.86 ± 0.13
Amplitude + sanitized phase	Unwrapping + linear trend removal	99.81 ± 0.26

## Data Availability

The RoboFiSense and RoboMNIST datasets used in this study are publicly available. The RoboFiSense dataset is available at https://github.com/SiamiLab/RoboFiSense (accessed on 1 February 2026). The RoboMNIST dataset is available from Figshare at https://doi.org/10.6084/m9.figshare.28179383.v1 (accessed on 1 February 2026). No new dataset was generated in this study. The source code for the proposed model will be made available upon publication.

## Abbreviations

The following abbreviations are used in this manuscript:

CSI	Channel State Information
Wi-Fi	Wireless Fidelity
ViT	Vision Transformer
BiVTC	Bidirectional Vision Transformer–Concatenated
MLP	Multi-Layer Perceptron
MHSA	Multi-Head Self-Attention
GAP	Global Average Pooling
CV	Cross-Validation
OFDM	Orthogonal Frequency-Division Multiplexing
RSSI	Received Signal Strength Indicator
